# Development and Implementation of an Intraoral Device for Occlusal Stability during Sports Performance: A Case Report

**DOI:** 10.3390/dj6040063

**Published:** 2018-11-08

**Authors:** Diana Silva, Joaquim Mendes, Jorge de Azevedo e Castro, Daniel Ferreira, André Moreira, Miguel Pais Clemente, Mário Vasconcelos

**Affiliations:** 1 Faculty of Dental Medicine, University of Porto, 4200-393 Porto, Portugal; up201402416@fmd.up.pt (J.d.A.eC.); mimd12064@fmd.up.pt (A.M.); miguelpaisclemente@hotmail.com (M.P.C.); 2 INEGI, Faculty of Engineering, University of Porto, 4099-002 Porto, Portugal; 3 Higher Institute of Health Sciences Egas Moniz, 2825-083 Monte de Caparica, Almada, Portugal; danielgcmferreira@gmail.com; 4 Biomaterials Department, Faculty of Dental Medicine, University of Porto, 4200-393 Porto, Portugal; mvasconcelos@fmd.up.pt

**Keywords:** intraoral devices, occlusal stability, sports dentistry, sports performance

## Abstract

Introduction: Sports dentistry assumes a clinical relevance, not only in the prevention of orofacial trauma by the use of mouthguards, but also with the development of intraoral devices that aim to provide greater occlusal stability, as well as a greater balance in the level of certain structures of the cranio-cervical-mandibular complex. In this way, the dentistry can have an intervention action on sports performance. Objectives: The objective of this research was to verify the existence of a correlation between occlusal stability and an eventual balance of some facial structures during sports performance using a specially developed Occlusal Stability Sports Performance Device. Methodology: An individualized mandibular intraoral device was manufactured and evaluated on an athlete canoeing. Infrared thermography was the complementary diagnostic method used for this purpose. Results: Greater symmetry of certain regions of interest of the cranio-cervico-mandibular complex was observed with the implementation of the Occlusal Stability Sports Performance Device. These areas were the anterior temporal muscle, the masseter muscle and the temporomandibular joint. No asymmetry decrease was found in the anterior triangle region of the neck. Conclusion: The use of this type of intraoral devices may allow greater occlusal stability and consequent balance of anatomical structures constituting the cranio-cervical-mandibular complex. Infrared thermography is an effective diagnostic tool for studying the results of the intraoral device on the cranio-cervico-mandibular complex use during canoeing.

## 1. Introduction

In dentistry, the physical problems related to sports practice that most occur are orofacial trauma, especially in contact sports. Periodontal disease, tooth erosion, and tooth decay may occur due to dehydration of the oral cavity during sports activity and frequent consumption of carbohydrates and acidic beverages, which together with the reduction of the saliva flow and consequent alteration of salivary properties can induce an alteration of the oral microbiome. There are also musculoskeletal and temporomandibular joint (TMJ) pathologies, derived from anxiety and stress situations associated to sports activity [1,2,3,4,5,6].

The TMJ is one of the most complex joints in our body and is classified as a “ginglymoarthrodial” joint since it is both, a ginglymus (hinging joint) and an arthrodial (sliding) joint. This joint consists of the mandibular condyle, the joint eminence, and the mandibular fossa of the temporal bone, with an articular disc between them. This joint has the function of linking the mandible to the skull and allowing the mandibular movements to be performed [7,8]. In conjunction with TMJ, there are other structures that form the cranio-cervical-mandibular complex (CCMC), namely the masticatory muscles, whose action besides mastication, swallowing and speech will permit the jaws to have a balanced function promoting the equilibrium with a perfect balance of the entire stomatognathic system. Thus, the CCMC activity may have repercussions on the musculature of the neck and chest region, and this functional interdependence may alter the individual’s posture [7].

Recently, one of the topics that was the subject of major interest for the sports dentistry scientific community was the prevention of orofacial trauma and structures, such as the TMJ, through the implementation of mouthguards that absorb and allow dissipation of impact forces [1,3,4,5,6].

Nevertheless, there are studies in the field of mouthguards that go beyond the topic of the prevention of orofacial trauma, promoting the occlusal stability through the use of an intraoral device. However, it may have repercussions regarding postural stability and a synergistic coordination of different muscular regions of the CCMC, as well as a consequent alteration in the neuromuscular pattern and stimulation of the sensory afferents of the trigeminal nerve, contributing to an improvement of the sport performance [9,10,11,12,13,14,15,16,17,18].

This paper presents a new intra-oral device manufactured by the authors, the Occlusal Stability Sports Performance Device (OSSPD), which was implemented in a canoeing athlete. In order to verify the possible correlation between occlusal stability through the use of this device and sports performance, infrared thermography was used. This is a non-invasive method that had been used as a complementary means of diagnosis regarding the equilibrium of specific anatomical regions like the masticatory muscles (masseter and temporal) [7,19].

## 2. Materials and Methods

The subject gave his informed consent for inclusion before he participated in the study. The study was conducted in accordance with the Declaration of Helsinki, and the protocol was approved by the Ethics Committee of Faculty of Dental Medicine of University of Porto (000522) on 5 February 2018. The inclusion criteria was that the individual should be a professional canoeing athlete, had the presence of all maxillary and mandibular teeth, had not been under orthodontic treatment, and did not report any symptomatology of temporomandibular disorders.

The OSSPD is an individualized mandibular intraoral device made from the thermoforming sheets of ethylene vinyl acetate (EVA). For the manufacture of the OSSPD, the impressions of the upper and lower arch were performed with Zhermack ortoprinth^®^ alginate, and the registration bite with silicone, Occlufast^®^, was taken in maximum intercuspidation.

After the impressions of the upper and lower jaws, the dental casts were obtained with gypsum stone type III. The excesses of the respective plaster models were removed to guarantee absence of interference in their articulation with the respective registration bite of each athlete.

**Figure 1:**
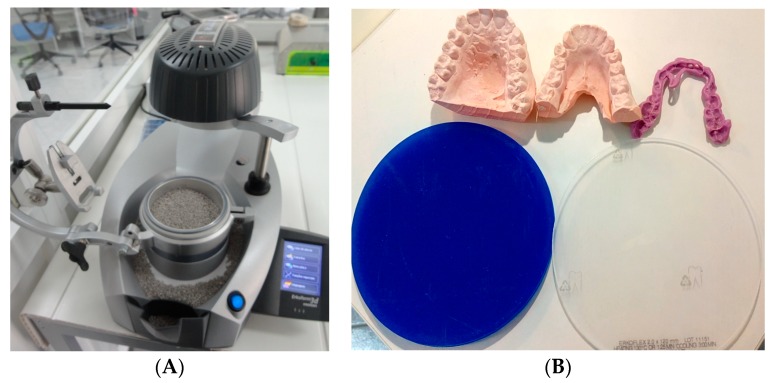
(**A**) Erkodent 3Dmotion, with the functions screen and respective program, at each stage of the Occlusal Stability Sports Performance Device (OSSPD) confection process, with parameters temperature, thickness, and cooling time. (**B**) Dental casts, registration bite and ethylene vinyl acetate (EVA) sheets of 2 mm and 4 mm (**left** to **right**), necessary for configuring OSSPD.

The equipment used for the manufacture of the OSSPD was the Erkodent 3Dmotion equipment (Erkodent, Pfalzgrafenweiler, Germany) (Figure 1A) and Erkodent (EVA) sheets 2 mm thick (diameter: 120 mm, Erkodent, Pfalzgrafenweiler, Germany) and 4 mm thick-diameter (Erkodent, Pfalzgrafenweiler, Germany): 120 mm (Figure 1B).

**Figure 2:**
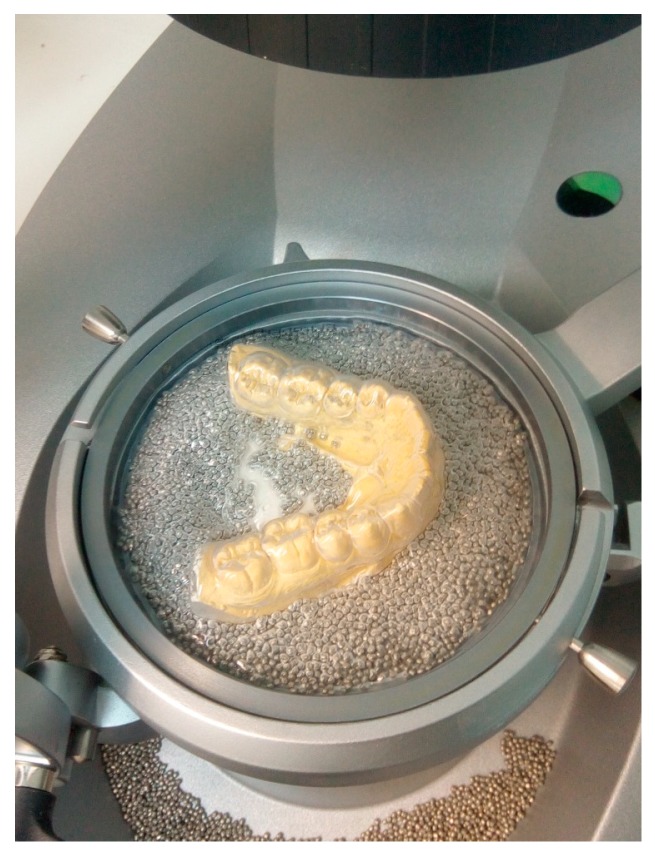
First lamination with 2 mm EVA foil.

The lower model was placed on the platform of the equipment, stabilized by the granules, and the upper model in the accessory Occluform-3D, for the first lamination with the sheet of EVA 2 mm, with isofoil, at a temperature of 130 °C and a cooling time of 3 min (Figure 2).

**Figure 3:**
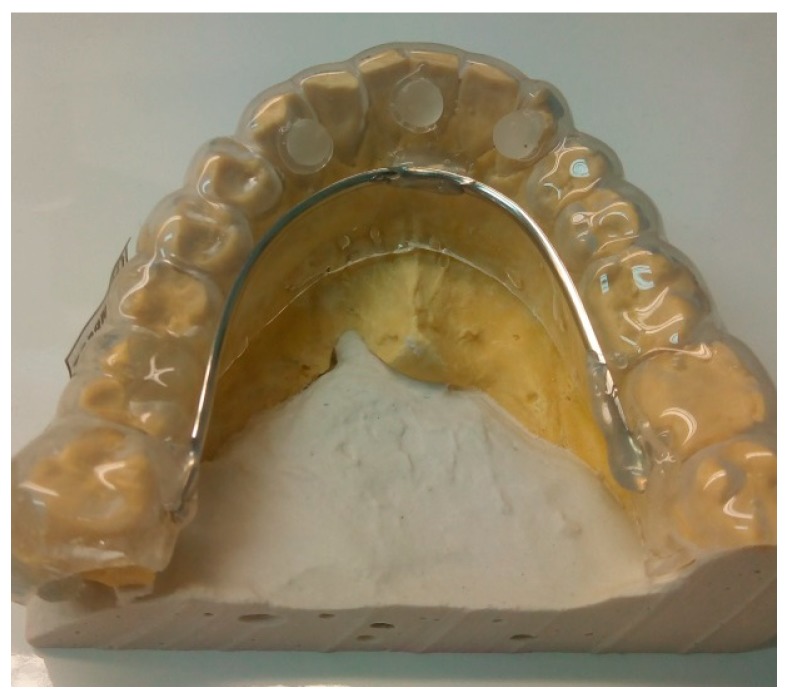
Reinforcement with wire steel and acrylic spheres.

After trimming and finishing the first lamination with a 2 mm EVA sheet, a reinforcement was prepared with a steel wire that was placed in the lingual zone of the intra-oral device, as well three acrylic spheres, one central and two lateral, were placed on the device (Figure 3).

**Figure 4:**
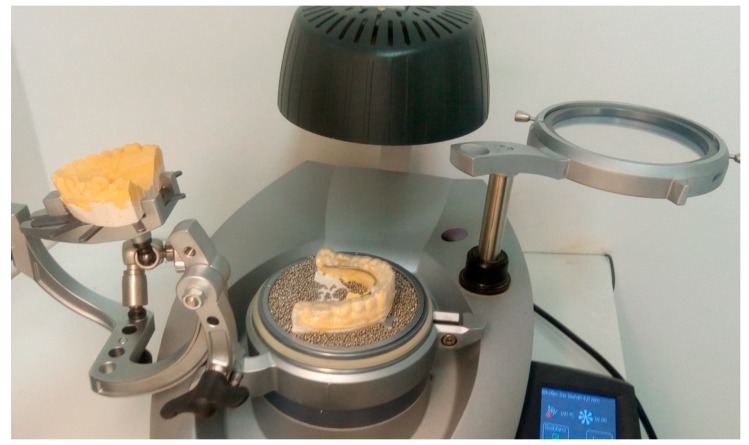
Preparation of the second lamination with 4 mm EVA foil.

Next, the second lamination was prepared with the 4 mm EVA sheet, without isofoil, and with previous articulation of the lower and upper casts according to the athlete’s registration bite. For the second lamination a temperature of 100 °C and 6 min of cooling was required (Figure 4).

Thus, the second lamination was carried out with the second plate of EVA of 4 mm thickness, on top of the first layer of EVA of 2 mm, the steel wire, and the acrylic spheres. During cooling, the upper dental cast was placed in the Occluform-3 accessory that was previously adjusted with the registration regarding the lower dental cast. This process had the intention to produce slight indentations on the occlusal surface of the second sheet of EVA.

**Figure 5:**
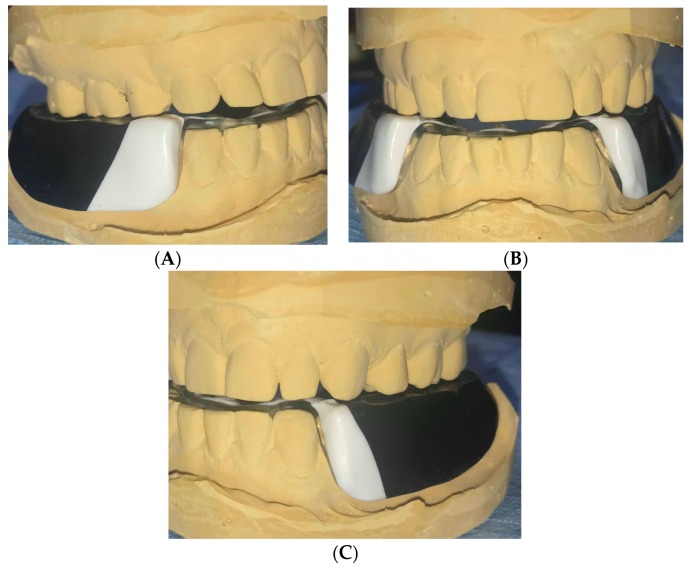
Dental casts and the OSSPD. (**A**) Right side view; (**B**) Frontal view; (**C**) Left side view.

Finally, all excess EVA of the OSSPD were cut out, including the anterior vestibular region from canine to canine, where the device measures about 1 mm, and in the posterior zone where the thickness was about 2–2.5 mm. The lingual and labial frenelum zones were cut using acrylic and micromotor drills. Polishing was done to smooth all edges (Figure 5).

## 3. Infrared Thermography Examination

In a second stage of the study, the effect of OSSPD on the CCMC was analyzed using infrared thermography. The athlete was asked to cut his beard to minimize the interference in the analysis of the regions of interest, as well as to avoid smoking or eating 2 h before the examination.

**Figure 6:**
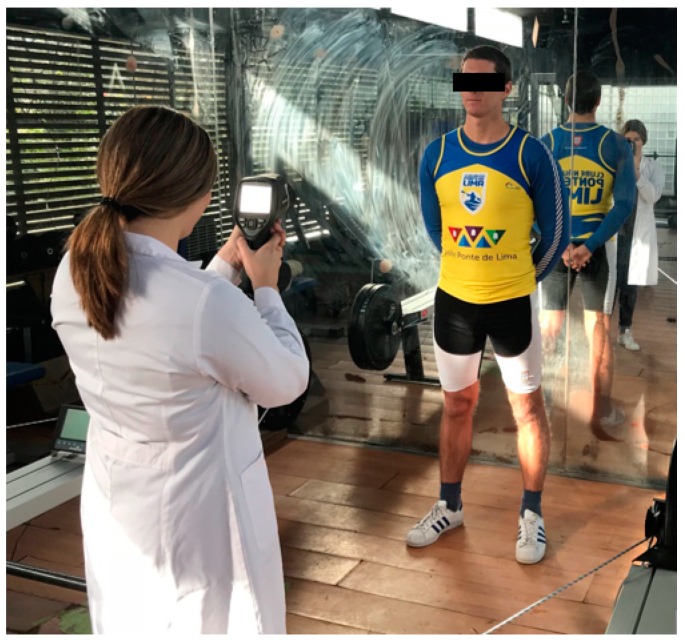
Positioning of the athlete for the acquisition of thermographic images.

The athlete rested for 20 min in a closed room where the canoeing exercises were to take place for acclimatization. The temperature (28.2 °C) and relative humidity (42%) were measured with the digital thermometer Testo 175H1. In this way, the necessary conditions for initiating the protocol of capturing the thermal images of the CCMC region were guaranteed (Figure 6).

**Figure 7:**
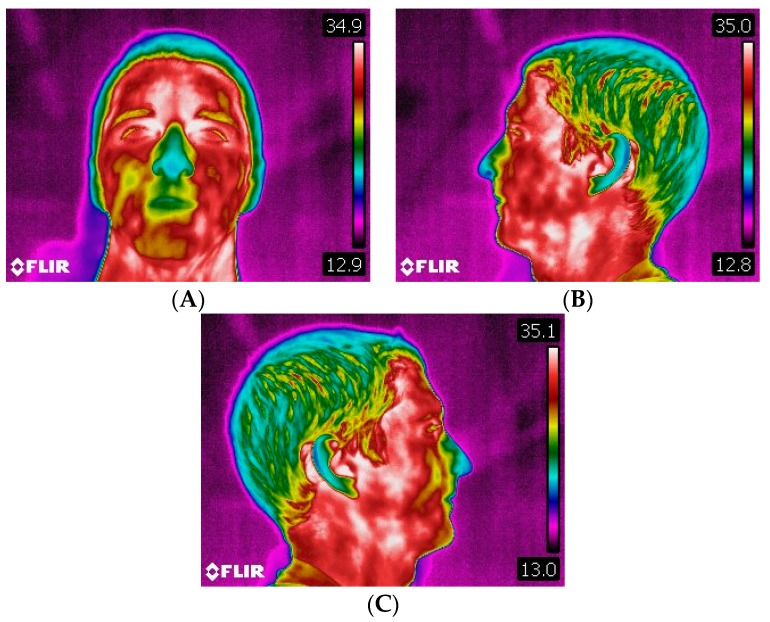
(**A**) Frontal thermogram. (**B**) Left side profile thermogram. (**C**) Right side profile thermogram.

**Figure 8:**
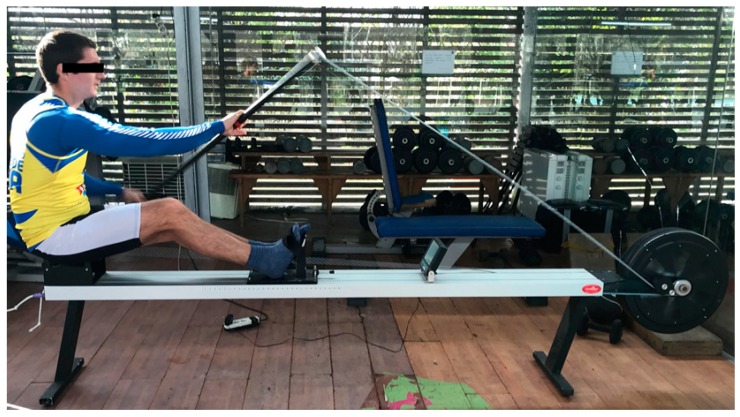
Ergometer for kayak simulation.

The thermography was performed with the athlete standing about 1.5–2 m from the thermographic camera, model FLIR^®^ E60sc (Flir Systems, Inc., Wilsonville, OR, USA, EUA), with the head erect, looking forward, and with lips at rest. Thermal images were obtained in three positions: frontal, left, and right lateral profiles. The images were taken by the same examiner, with the same distances between the athlete and the camera operator. The initial thermograms of the face were obtained at rest position (Figure 7). Afterwards, the standardized sports series were performed on a kayak: 1 min at 11 km/h; 2 min at 12–12.5 km/h; 1 min cool down (at least 8 km/h); 1 min at 13.5 km/h; 1 min cool down (at least 8 km/h); 0.5 min at 14.5 km/h. The respective series were performed on the Dansprint^®^ ergometer (Dansprint, Hvidovre, United Kingdom), which simulates the movement of the kayak (Figure 8).

**Figure 9:**
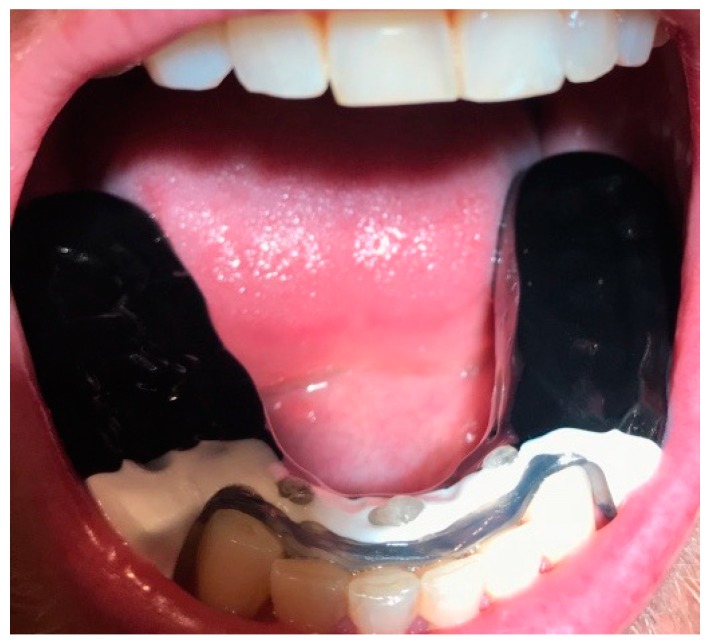
Occlusal view of OSSPD, in the oral cavity.

Immediately at the end of the standardized series, new face infrared thermograms (front, right, and left profile) were performed in the same room. Then, the intraoral device, OSSPD, was placed in the mouth in order to verify the need for occlusal adjustments (Figure 9).

The device was adjusted using an acrylic drill, mounted on a handpiece and micromotor, after recording the occlusal contacts using articular paper. The spheres were placed in order to provide a sensory-motor stimulus of the trigeminal nerve, more precisely the mandibular branch. 

**Figure 10:**
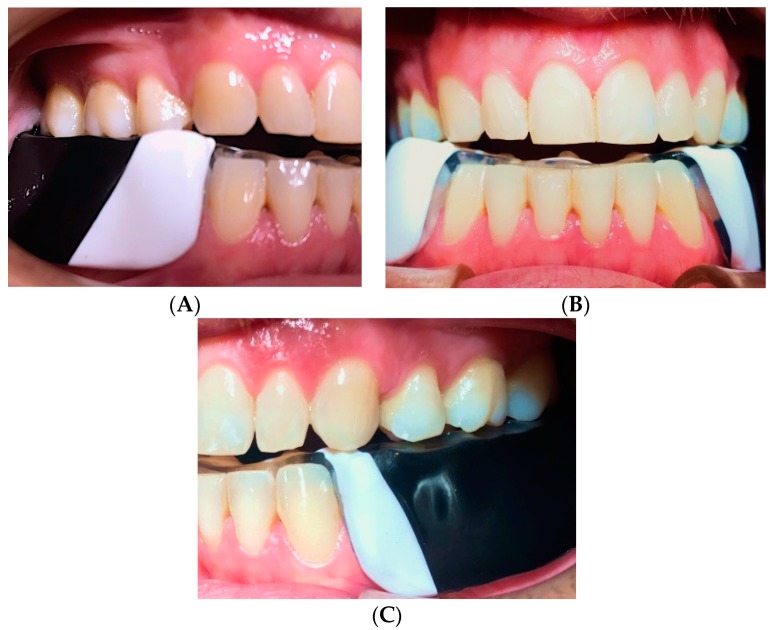
(**A**) right side view of OSSPD in mouth; (**B**) anterior view of the OSSPD in the mouth; (**C**) left lateral view of OSSPD in the mouth.

A new thermographic recording of the CCMC was performed (frontal, right profile, left profile) after a rest period of 20 min. Then, the device was placed in the athlete’s mouth, already adjusted and adapted (Figure 10). It is worth noting that not all intraoral devices have to be adjusted; most of them come from the laboratory and allow a perfect adaptation to the oral cavity of the athletes. However, it is important to avoid interferences between the OSSPD and the upper jaw. The occlusal adjustment was made so that no discomfort or any disadvantage could arise for the athlete when using this new intraoral OSSPD.

Then, the same standardized series of the canoeing exercises were repeated and the infrared thermography of the CCMC was again performed (front, right, and left profile).

## 4. Thermographic Analysis

**Figure 11:**
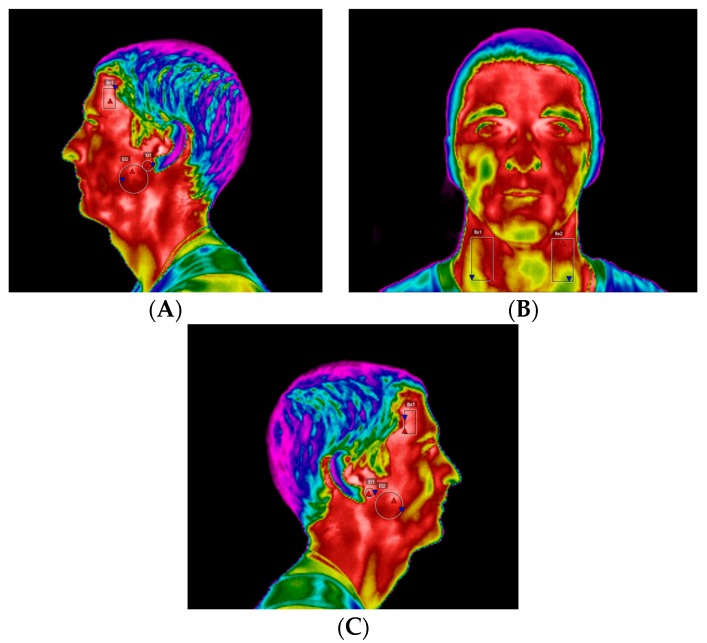
Regions of interest of the thermographic analysis: Anterior neck triangle, temporomandibular joint (TMJ), superficial masseter and anterior temporal muscles. (**A**) Left profile; (**B**) Front view; (**C**) Right profile.

The respective thermographic images were analyzed using FLIR Tools 6.2, always by the same examiner, in order to compare the temperature differentials with respect to the initial situation, with and without OSSPD, in four regions of interest on the CCMC, namely TMJ, superficial masseter muscle, anterior temporal muscle, and anterior neck triangle (Figure 11). The regions of interest were studied on the lateral side of each area, that is, the left and right side of each zone under study of the athlete.

The asymmetry to the contralateral region of the CCMC was analyzed for each region of interest.

## 5. Results

**Figure 12:**
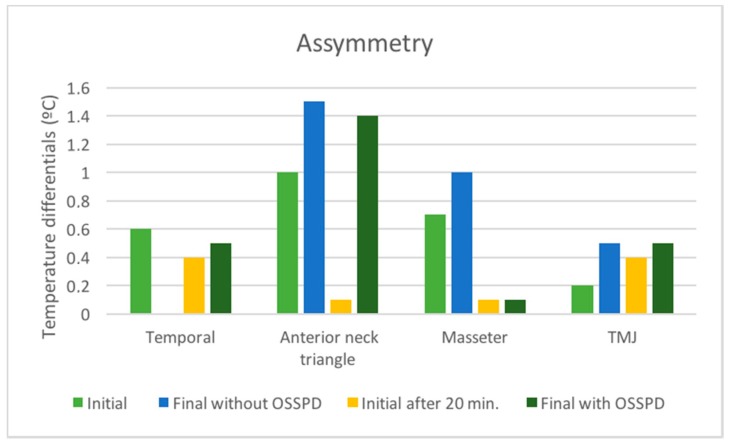
Asymmetry value of contralateral regions of interest (ROIs) in the different times of capture of the thermographic images, initial, final without OSSPD, after the rest of 20 min, and final with OSSPD for the four regions of interest: TMJ, anterior temporal, masseter, and anterior neck triangle.

Figure 12 shows the asymmetry values obtained between the right and left sides of the CCMC, in the different capture times: initial, final without OSSPD, initial after of 20 min., and final with OSSPD for the four regions of interest (TMJ, anterior temporal, masseter, and anterior neck triangle).

## 6. Discussion

The results of this research are intended to complement an area where there is still little scientific validated information regarding the use and effect of intraoral devices that aim for greater occlusal stability during sports performance.

The results show that there is a decrease in the asymmetry for the TMJ, masseter, and anterior temporal region after the implementation and use of the OSSPD by the canoeing athlete [20]. There is also a greater approximation of values with regard to asymmetry with OSSPD, respectively, 0.1 °C, 0 °C, and 0.1 °C, which is the result of a greater equilibrium and harmonious balance of certain muscular groups that constitute the CCMC.

In this way, one of the main objectives proposed with the idealization of OSSPD was visible in this athlete, by showing the inherent occlusal stability obtained through the intraoral device. This situation led to a balance in the forces exerted by some of the CCMC muscles, which induced a new neuromuscular pattern and a greater symmetry in the distribution of the force applied between the observed regions on the right and the left sides of the CCMC.

Regarding the anterior triangle of the neck, although there was an increase in the asymmetry, it is possible to ascertain that the asymmetry of the final moment without OSSPD compared with OSSPD decreased 0.1 °C, meaning that despite a less representative improvement, the OSSPD also influenced this region of the CCMC.

The OSSPD is approximately 2–2.5 mm thick, from the premolar region to the distal zone of the second molar, thus seeking to respect the neutral zone and not interfere with the possible increase of muscular hyperactivity. On the occlusal surface of the device, slight indentations of the maxillary position were made in order to allow the jaw to easily find a natural position in relation to the antagonist arch, which also was intended to allow better comfort, as was also explained by Zupan, M (2018) [21].

On the anterior lingual region, three acrylic spheres were placed in order to increase the proprioceptive component and to explain the neurophysiological function with regard to the stimulation of the cranial nerves and regulation of the autonomic nervous system, in view of a better neuromuscular balance of CCMC.

Cranial nerve V, the cranial trigeminal nerve, is divided into three branches, ophthalmic, maxillary, and mandibular, and has mixed function, that is, its effects are felt at both the motor and sensory levels. The sensory function is present through the transmission of afferent sensory impulses of the tongue, masticatory muscles, mandibular and TMJ reflexes, and also with regard to the coordination of mastication, as described by Woźniak, 1982 et al. and Willis, 1979. Parallel to this, Dessem, 1999, suggested that the proprioceptive feedback captured through the trigeminal nerve reaches the cervical region, and therefore is a coordination between muscle activity of several muscle chains that constitute the CCMC. In addition, some authors argue that the nerve endings of the tongue are also related to the positioning of the head, given the interconnection between the twelfth cranial nerve, the hypoglossal nerve, and the first three cervical nerves (C1, C2, C3) that enervate muscles of the CCMC, such as sternocleidomastoid and trapezius, as advocated by Gestreau et al., 2005 and Vuillerme et al., 2008 [22,23,24,25,26].

On the other hand, the interest of associating infrared thermography as a complementary means of diagnosis and therapy in the validation of this study was obtained on the analysis of a professional kayak athlete after the implementation of the OSSPD. Previous studies, in which thermography was related to posture, suggest its usefulness in this same mode of canoeing, as Silva, 2018, points out [19]. The advantages of infrared images are also recognized in the diagnosis and therapeutics in temporomandibular disorders, in which the results obtained with the acquisition of thermographic images are reliable in the evaluation of the changes felt at the level of the CCMC, corroborating with Gabriel, 2016 [7].

This study presents some limitations due to the size of the sample, and there is still a long way go to recognize the causal effects of the acrylic spheres placed on the lingual zone of the intraoral device and the sensory-motor stimulus of the trigeminal nerve. Many compensation mechanisms occur within the neuromuscular system to regulate body balance, and a systematic review concluded that the available posturographic techniques and devices have not consistently found any association between body posture and dental occlusion [27].

## 7. Conclusions

The development of this new intraoral device proved to be effective as it was possible to obtain an occlusal stability in the athlete during his sports practice.

The use of thermography as a diagnostic and therapeutic method proved adequate for the study of different constituent structures of CCMC, before and after the practice of the modality in question. Although the sample is small, since this is a preliminary report of the development of a new intra-oral device, the analysis of the thermograms showed that the use of the OSSPD allowed a greater balance of the temporomandibular joint, anterior temporal muscle, and masseter muscle. The action of this new intraoral device was important not only at the level of the occlusal component, but also in the promotion of a greater neuromuscular balance.

The OSSPD may be a promising device in the field of sports dentistry, where the dentist may have an active role in perceiving the individual need of each athlete, resulting from the practice of the modality in question, in order to allow a better sports performance.
